# Upregulated circRNA_102231 promotes gastric cancer progression and its clinical significance

**DOI:** 10.1080/21655979.2021.1960769

**Published:** 2021-08-10

**Authors:** Gaofeng Yuan, Wenwen Ding, Bingjie Sun, Lin Zhu, Yuewen Gao, Lulu Chen

**Affiliations:** a Department of Oncology, The AffiliatedSuqian First People's Hospital of Nanjing Medical University, Suqian, Jiangsu, PR China; b Department of Surgery, The People’s Hospital of Rizhao City, Rizhao, Shandong, PR China; c Disinfection Supply Center, The AffiliatedSuqian First People's Hospital of Nanjing Medical University, Suqian, Jiangsu, PR China; d Department of General Surgery, The People’s Hospital of Rizhao City, Rizhao, Shandong, PR China

**Keywords:** CircRNA_102231, IRTKS, gastric cancer, biomarker, tumorigenesis

## Abstract

Circular RNAs (circRNAs) are a type of endogenous non-coding RNAs implicated in cancer progression. This study explored the expression levels, clinical implication and possible molecular mechanism of circRNA_102231 in gastric cancer (GC). Gene Expression Omnibus (GEO) was used to analyze differentially expressed circRNAs. CircRNA_102231 expression was verified by qRT-PCR in GC tissues and plasma. The effects of circRNA_102231 was tested by CCK-8, colony formation, EdU and Transwell assays and xenograft tumor model. RNA pull-down and immunoprecipitation (RIP) assays were used to analyze the interaction between circRNA_102231 and IRTKS. CircRNA_102231 expression was significantly upregulated in GC tissue and plasma samples, which can be used as a biomarker for GC diagnosis and prognosis. The function assays showed that circRNA_102231 knockdown inhibited GC cell proliferation and invasion both *in vitro* and *in vivo*. CircRNA_102231 was able to bind to IRTKS, increasing IRTKS protein stability, leading to GC progression. Overexpression of IRTKS effectively rescued the reduced cell viability and invasion caused by silencing of circRNA_102231. In sum, our data demonstrate that circRNA_102231 is a novel oncogene in GC and acts as a potential biomarker and therapeutic target for GC patients.

Abbreviations

circRNAs: circular RNAs; GC: gastric cancer; GEO: Gene Expression Omnibus; RIP: RNA immunoprecipitation; DEGs: differentially expressed genes

## Introduction

Gastric cancer (GC) remains a major cancer worldwide and will cause over 1 million new cases and an estimated 769,000 deaths by 2020 (equivalent to 1 in 13 deaths worldwide), ranking fifth in morbidity and fourth in mortality worldwide [1]. Endoscopy combined with enhanced CT is currently the gold standard for the diagnosis of early GC, but this method is invasive and cannot be used as a long-term method for the diagnosis and monitoring of tumor progression [2]. Despite the good progress in GC treatment in recent years, the overall survival rate is still unsatisfactory [3]. Therefore, it is of great significance to search for new diagnostic markers and treatment strategies.

Circular RNA (CircRNA) is a new type of noncoding RNA widely expressed in eukaryotes, characterized by a covalently closed ring structure [4]. Previous researchers thought that it is a by-product or the result of wrong splicing in the process of precursor mRNA processing, which has no biological significance and has not attracted enough attention [5]. With the development of high-throughput RNA sequencing technology and bioinformatics, the role of circRNA is emerging, with important biological functions such as cell signal transduction, protein folding and translation [6]. Abnormal expression of circRNA affects the occurrence and development of many kinds of human tumors [7]. For example, circSETD3 was significantly increased in nasopharyngeal carcinoma, knockdown of circSETD3 inhibited cell proliferation and invasion [8]; circDNMT1 was highly expressed in breast cancer cells and tissues, it enhanced breast cancer progression by activating autophagy [9]; likewise, in hepatocellular carcinoma, circ_0008274 was shown to be an oncogene via facilitating cell proliferation and metastasis both *in vitro* and *in vivo* [10]. And circ-LRP6 promoted esophageal squamous cell cancer progression via regulation of Myc oncoprotein [11].

To identify the key circRNAs in GC, we analyzed Gene Expression Omnibus (GEO) database, GSE152309, GSE141977 and GSE121445, containing circRNA high-throughput sequencing data from GC and paracancerous tissues. The intersection results showed that circRNA_102231 had the largest differential expression multiple, and was elevated in GC as compared to normal tissues. Then, we verified the upregulation of circRNA_102231 in our collected GC tissues and plasma, and further explored its biological function and possible molecular mechanism. Therefore, our data aimed to reveal the functional, prognostic, and predictive roles of circRNA_102231 in GC, which may bring new targets for clinical diagnosis and treatment of GC.

## Methods

### Analysis of GEO data

The raw data of GSE152309, GSE141977 and GSE121445 were downloaded from GEO database and analyzed using R language. The affy package was used for background correction and data normalization. The Limma package was used to screen differentially expressed genes (DEGs). The cutoff value was |log2FC| >1 and FDR <0.05 (FC, fold change; FDR, false discovery rate). The intersection result of three GEO datasets showed that circRNA_102231 had the largest differential expression multiple.

### GC tissues and plasma

A total of 120 pairs of GC and adjacent normal tissues were collected from The People’s Hospital of Rizhao City, all of which were pathologically diagnosed as GC. The excised tissues were immediately stored in liquid nitrogen. The clinical data were collected for analyzing their correlations with circRNA_102231. In addition, the plasma samples from 48 GC patients and 33 healthy controls were also obtained for assessing the diagnostic value of plasma circRNA_102231. Each human participant signed informed consent for the publication of their clinical information and clinical details. This study was approved by the ethics committee of People’s Hospital of Rizhao City and complied with the principles of the Declaration of Helsinki (as revised in 2013).

### qRT-PCR analysis

Total RNA extraction was conducted using Trizol reagent (Invitrogen, USA), followed by cDNA synthesis by using Revertaid Reverse Transcription Kit (Thermo Fisher Scientific, USA) as per manufacturer’s instructions. Gene quantification and amplification were conducted using Absolute qPCR Premix (Thermo Fisher Scientific) with StepOnePlus™ system. GAPDH was utilized as reference control calculating gene relative expression. The primer sequences are shown below:

circRNA_102231: F: 5`-TTCTGAGGACAGACAGGTGCT-3`, R: CGGTGAACTGACCTGTACCC;

IRTKS: F: TGTGGAGACCGTTACTTCTCG, R: AAGCCACAGTGCTTATCAACC;

GAPDH: F: ACCCAGAAGACTGTGGATGG, R: TTCAGCTCAGGGATGACCTT.

### Cell culture and transfection

AGS and MKN45 cells were purchased from ATCC, and cultured in DMEM medium supplemented with 10% FBS. Cells were tested regularly every month for mycoplasma contamination. Cell transfection was performed using Lipofectamine 3000 (Invitrogen) manufacturer’s instructions.

### Lentiviral-mediated circRNA_102231 knockdown

To silence circRNA_102231, three siRNAs against the junction sequence of circRNA_102231 were designed and synthesized by GENECHEM (Shanghai, China), followed by transfection into GC cells and qRT-PCR verification. The one with the best silence efficiency (si-circRNA_102231#1, 5`-GTTCACAGGCCTCTGGCTGTA-3`) was used to insert into hU6-MCS-CBh-gcGFP-IRES-puromycin lentiviral vector (GENECHEM). After 72 h, green fluorescence was observed under a microscope, and stable cell clones were screened using puromycin.

### Cell function assays

These assays were conducted according to previous protocols [12]. In brief, for cell colony assay, 800 cells were plated onto six-well plates and cultured for 14 days. Cells were then stained with crystal violet. DNA synthesis rate was tested by EdU Staining Detection Kit provided by RiboBio (Guangzhou, China). The number of stained positive cells represents the rate of DNA synthesis. Cell viability was tested by CCK-8 assay, 1000 GC cells were seeded onto 96-well plates and cultured for the indicated time. Then, the commercialized CCK-8 solution (Dojindo, Kumamoto, Japan) was added into each well and incubated for 2 h. The absorption value at 450 nm was tested by a microplate reader. Cell invasion was detected by Transwell assay, briefly, GC cells were plated onto Transwell chamber. After incubation for 24 h, the invaded cells were stained with crystal violet.

### RNA pull-down and immunoprecipitation (RIP)

For RNA pull-down assay, the biotin-labeled circRNA_102231 probe was designed and synthesized by RiboBio, and verified by qRT-PCR analysis. GC cells were treated with Co-IP buffer; then, cell lysates were collected and added with above circRNA_102231 probe at 4°C overnight. After incubation with PuriMag G-streptavidin magnetic beads at room temperature for 1 h. The magnetic beads were washed and eluted by SDS loading buffer at 100°C for 5 min, followed by western blot analysis. RIP assay was conducted based on previous protocol [13] by using Magna RIP™ RNA-Binding Protein Immunoprecipitation Kit according to manufacturer’s instructions (MERCK, USA).

### Western blot

Total protein was collected by using RIPA lysis buffer supplemented with protease inhibitor cocktail tablet and phosphatase inhibitors (Roche, USA). Afterward, cells were centrifuged at 14,500 rpm for 10 min at 4°C. Supernatant was employed for protein loading. Then, the proteins were electro-transferred to a PVDF membrane and blocked using 5% nonfat milk powder. After incubation with anti-IRTKS (ab226344, Abcam) and Goat Anti-Rabbit IgG H&L (ab6721, Abcam), the membrane was developed using the Chemiluminescent Substrate Reagent Kit (Invitrogen).

### Xenograft tumor model

MKN45 cells with or without circRNA_102231 knockdown were subcutaneously injected into nude mice, four in each group. After 28 days of growth in specific pathogen-free (SPF) condition, all mice were sacrificed, and tumor tissues were collected and weighted, followed by qRT-PCR and western blot assays. All investigations involving animals adhered to the institutional guidelines for animal welfare from the Institutional Animal Care and Use Committee of People’s Hospital of Rizhao City.

### Statistical analysis

Comparison between the two groups was performed by either the Student’s t-test or the Chi-square test. Kaplan–Meier method was used to generate survival curve, which was analyzed by Log-rank test. Receiver operating characteristic (ROC) curve was used to analyze the diagnostic utility of plasma circRNA_102231. Different cutoff values, *p*< 0.05 (*), *p*< 0.01 (**) and *p*< 0.001 (***), were considered significant.

## Results

In the current study, we conducted a series of functional and *in vivo* assays to explore the tumor-promoting effect of circRNA_102231. More importantly, we also revealed the underlying mechanism of circRNA_102231, it could be a protein binding partner for IRTKS, a well-documented oncogene in GC, and prevented IRTKS protein from degradation. Pre-clinically, we found that circRNA_102231 was a promising diagnostic and prognostic biomarker for GC patients.

### CircRNA_102231 is highly expressed in GC tissues and plasma samples

**Figure 1.**
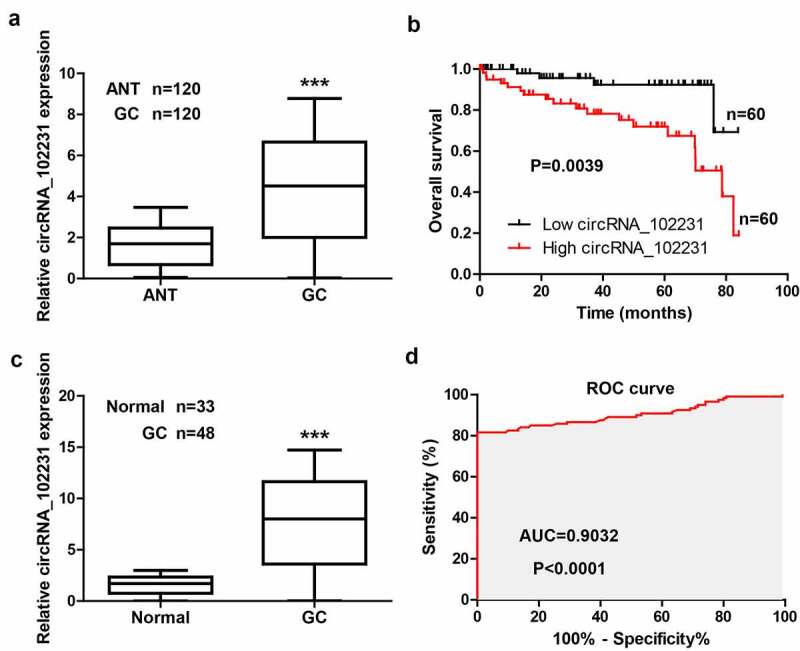
CircRNA_102231 is upregulated in GC. (a). qRT-PCR analysis of circRNA_102231 expression in paired GC and normal tissues. (b). The survival curve of GC patients based on median circRNA_102231 level in GC tissues. (c, d). qRT-PCR analysis of plasma circRNA_102231 expression, followed by ROC curve analyzing its diagnostic value. ***p< 0.001. ANT = adjacent normal tissue

We tested the level of circRNA_102231 in 120 paired GC and normal tissues, the qRT-PCR results showed that circRNA_102231 was significantly increased in GC tissues (Figure 1(a)). As shown in Table 1, high expression of circRNA_102231 was observed more often in patients with larger tumor volume, lymph node metastasis and higher clinical stage. Moreover, Kaplan–Meier plotter showed that GC patients with high circRNA_102231 had shorter survival time than those with low circRNA_102231 (Figure 1(b)). Further, we collected the plasma samples to test the diagnostic value of circRNA_102231, the results showed that circRNA_102231 expression in the plasma of GC patients was about 8-fold higher than that of healthy controls (Figure 1(c)), with an area under ROC (AUC) was 0.9032 (Figure 1(d)).

**Table 1.** Correlation between circRNA_102231 expression and clinicopathological features in GC patients (n = 120)

Parameters	All cases	circRNA_102231 expression		*P* value
		Low (n = 60)	High (n = 60)	
Gender				
Male	75	40	35	0.346
Female	45	20	25	
Age (years)				
≤ 60	51	30	21	0.097
> 60	69	30	39	
Tumor size				
≤ 5	72	42	30	0.025
> 5	48	18	30	
Lymph node metastasis				
No	68	42	26	0.003
Yes	52	18	34	
TNM stage				
I–II	70	42	28	0.011
III–IV	50	18	32	
Differentiation grade				
Well/moderate	65	35	30	0.361
Poor	55	25	30

### CircRNA_102231 knockdown reduced GC cell proliferation and invasion

**Figure 2.**
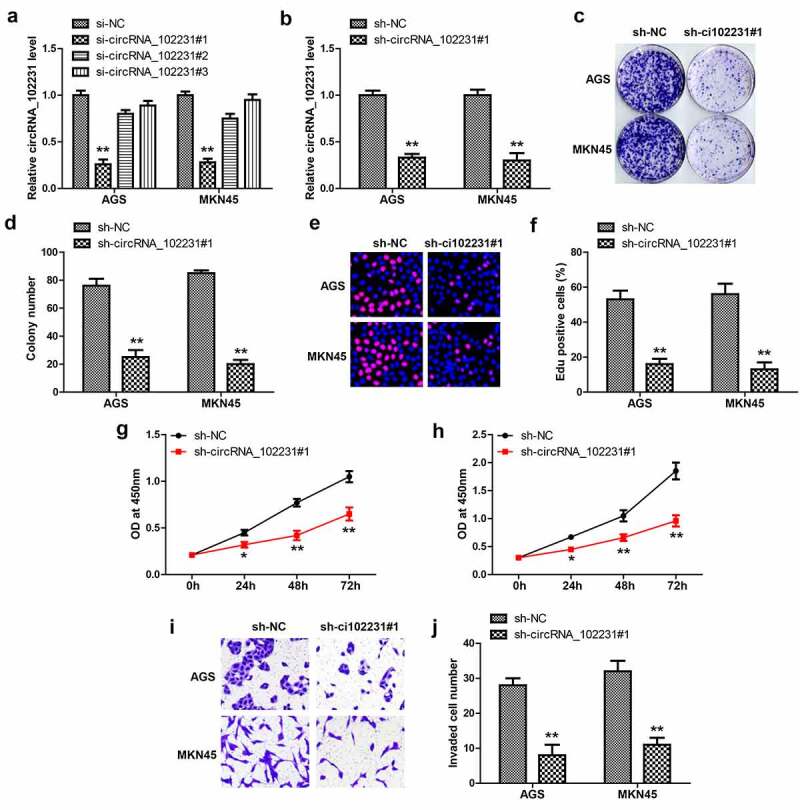
Knockdown of circRNA_102231 inhibits GC progression. (a, b). qRT-PCR analyzing the knockdown efficiency of these siRNAs. (c–j). Colony formation, EdU, CCK-8 and Transwell assays in GC cells after circRNA_102231 knockdown. *p< 0.05, **p< 0.01

Then, we designed three siRNAs targeting circRNA_102231 junction site to silence circRNA_102231 (Figure 2(a)), the one with the best silence efficiency was used to generate stable GC cell lines (Figure 2(b)). The plate cloning and EdU assays showed that colony formation (Figure 2(c,d)) and DNA synthesis rate (Figure 2(e,f)) were significantly reduced after circRNA_102231 knockdown. Moreover, cell viability was also weakened by silencing of circRNA_102231, as demonstrated by CCK-8 assays (Figure 2(g,h)). Transwell assays showed that circRNA_102231 knockdown caused fewer cells to cross the stromal membrane (Figure 2(i,j)).

### CircRNA_102231 binds to IRTKS

**Figure 3.**
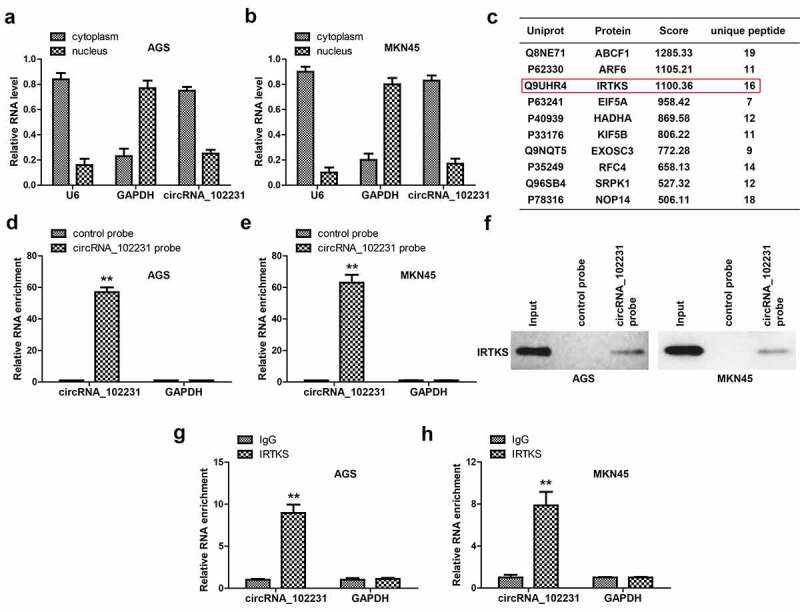
CircRNA_102231 interacts with IRTKS. (a, b). qRT-PCR analysis of the location of circRNA_102231. C. The top ten proteins pulled down by circRNA_102231. (d–f). qRT-PCR verifying the specificity and effectiveness of circRNA_102231 probe, followed by western blot assay. G, H. RIP assay testing the enrichment of circRNA_102231 by anti-IRTKS. **p< 0.01

To identify the possible mechanism of circRNA_102231, we first tested its location in GC cells. The qRT-PCR results showed that about 80% of circRNA_102231 was located in the cytoplasm (Figure 3(a,b)). Given that circRNA is able to function via direct interaction with functional proteins, we performed RNA pull-down coupled mass spectrum analysis. As shown in Figure 3(c), some proteins were pulled down by circRNA_102231, IRTKS attracted our attention because of its importance in promoting the progression of GC [14]. We verified the specificity and effectiveness of circRNA_102231 probe in GC cells (Figure 3(d,e)), and western blot showed that circRNA_102231 assuredly enriched endogenous IRTKS in both AGS and MKN45 cells (Figure 3(f)). Reciprocally, RIP assay showed that circRNA_102231 was enriched by anti-IRTKS, not by anti-IgG (Figure 3(g,h)).

### CircRNA_102231 enhances IRTKS protein stability

**Figure 4.**
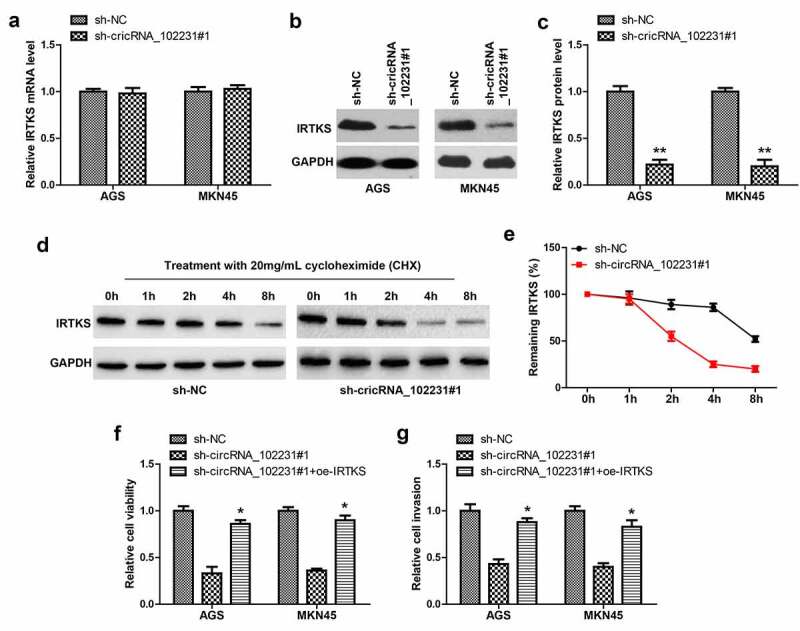
CircRNA_102231 increases IRTKS protein stability. (a). qRT-PCR analysis of IRTKS mRNA level in circRNA_102231-silenced GC cell lines. (b, c). Western blot testing IRTKS protein in circRNA_102231-silenced GC cell lines. (d, e). Western blot testing the effect of circRNA_102231 on IRTKS protein stability. (f, g). Cell viability and invasion assay in circRNA_102231-silenced GC cell lines transfected with IRTKS-expressing vector. *p< 0.05, **p< 0.01

Next, we tested whether circRNA_102231 affected IRTKS level, as shown in Figure 4(a), IRTKS mRNA remained unchanged after circRNA_102231 knockdown; while IRTKS protein was substantially decreased (Figure 4(b,c)). Then, we treated GC cells with cycloheximide (CHX), western blot showed that silencing of circRNA_102231 markedly shortened the half-life period of IRTKS protein (Figure 4(d,e)). Functionally, the reduced cell viability and invasion caused by circRNA_102231 knockdown were significantly rescued by IRTKS overexpression (Figure 4(f,g)).

### *Knockdown of circRNA_102231 reduces tumor volume* in vivo

**Figure 5.**
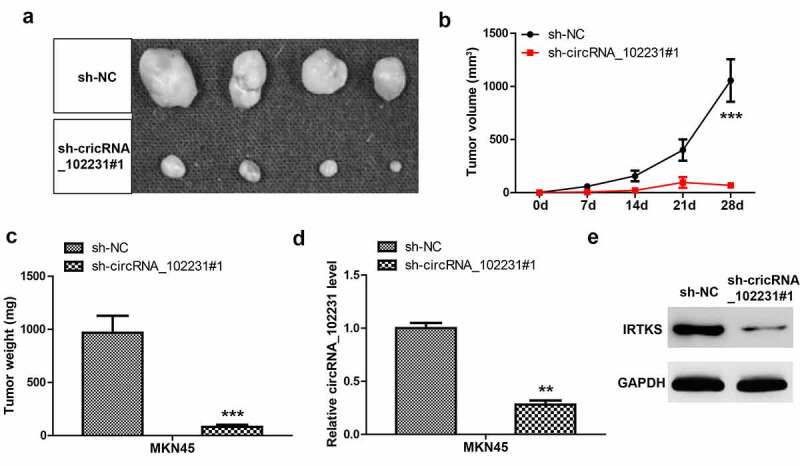
CircRNA_102231 depletion inhibits tumor growth. (a–c). Tumor image, volume and weight in control and circRNA_102231-silenced groups. (d, e). qRT-PCR and western blot assays analyzing circRNA_102231 and IRTKS protein level, respectively. **p< 0.01, ***p< 0.001

Lastly, we explored the *in vivo* effect of circRNA_102231, the nude mice were subcutaneously injected with circRNA_102231-depleted MKN45 cells. Twenty-eight days later, all mice were sacrificed and the results showed that tumor volume and weight developed by circRNA_102231-depleted cells were significantly less than those developed by control cells (Figure 5(a–c)). Moreover, we verified the knockdown of circRNA_102231 in tumor tissues developed by circRNA_102231-depleted MKN45 cells (Figure 5(d)). Consistently, IRTKS protein level was reduced after circRNA_102231 silencing *in vivo* (Figure 5(e)).

## Discussion

In the present study, we for the first time describe the biological of circRNA_102231 in GC. High circRNA_102231 was observed in GC tissues and plasma, it promoted GC cell proliferation and invasion by interacting with oncoprotein, IRTKS. Importantly, we found that circRNA_102231 served as a protein stabilizer, which increased the half-life period of IRTKS protein, thereby prolonging its cancer-promoting effect. Hence, our data underscore the importance of circRNA_102231 in GC tumorigenesis, and also provide new insights into the pathogenesis of GC.

Improving the diagnosis of GC will bring great benefits to the prognosis of clinical patients, some markers have also been used in clinical practice, such as CEA and CA-199 [15]. However, the sensitivity and specificity of CEA and CA-199 in serological detection of early GC were not high [16], it is particularly important to find more specific markers. The special structure of circRNA makes it easy to obtain, high sensitivity, strong specificity and not easy to degrade [17,18]. Compared with traditional assays, circRNA is more in line with the criteria for early diagnosis and prognosis evaluation of human cancer [19,20]. For instance, CDR1as, a circRNA of great concern, was significantly upregulated in various human solid tumors, which has been identified as a reliable diagnostic and prognostic biomarker with high accuracy and efficiency by meta-analysis [21]. And a recent study generated a four-circRNA-based cirScore to predict postoperative recurrence in stage II/III colon cancer [22]. Besides, circRASSF2 was significantly increased in breast cancer, and predicted a poorer overall and progression-free survival of breast cancer patients [23]. Herein, we found that circRNA_102231 was highly expressed in GC tissues, which was closely correlated with poor outcome, suggesting that circRNA_102231 may be a promising prognostic indicator for GC patients. Moreover, high circRNA_102231 was also found in GC plasma, the AUC exceeded 0.9, indicating its reliable noninvasive diagnostic value. Without doubt, future multi-center large-scale investigation is needed to verify the diagnostic and prognostic value of circRNA_102231 in GC.

Emerging evidence suggests that circRNA can bind to different proteins to form specific circRNA protein complexes, controlling protein function, subcellular localization or turnover [24,25]. In this study, by using RNA pull-down coupled mass spectrum analysis, we found that circRNA_102231 was capable to directly bind to IRTKS, a well-characterized oncogene in GC that promotes p53 ubiquitination and degradation via E3 ubiquitin ligase MDM2 [14]. Furthermore, circRNA_102231 stabilized IRTKS protein, specifically, extended its half-life period from about 2 h to 8 h, which amplified its cancer-promoting effect at the time level. Importantly, IRTKS overexpression rescued the attenuated cell malignant phenotype caused by circRNA_102231 knockdown, indicating the axis of circRNA_102231/IRTKS does exist in GC. Therefore, we reveal the function of circRNA_102231 as the protein-binding partner, meanwhile, sheds light on the reason for the high expression of IRTKS in GC, which may be due to circRNA_102231 upregulation. Further studies are needed to clarify how circRNA_102231 increases the stability of IRTKS at the post-translational modification level, which may be related to acetylation, phosphorylation or ubiquitination, etc.

## Conclusions

Our findings expound the carcinogenic effect of circRNA_102231 in GC, and provide a promising indicator for GC diagnosis and prognosis, which raises the possibility of considering it as a potential target for cancer therapy.

## Highlights

1. CircRNA_102231 is highly expressed in GC tissues and plasma.
2. Knockdown of circRNA_102231 reduces GC cell malignant behaviors.
3. CircRNA_102231 binds to IRTKS protein and enhances its stability.
4. CircRNA_102231 knockdown inhibits tumor growth *in vivo*.
